# Witches’ broom resistant genotype CCN51 shows greater diversity of symbiont bacteria in its phylloplane than susceptible genotype catongo

**DOI:** 10.1186/s12866-018-1339-9

**Published:** 2018-11-23

**Authors:** Juliano Oliveira Santana, Karina Peres Gramacho, Katiúcia Tícila de Souza Eduvirgens Ferreira, Rachel Passos Rezende, Pedro Antônio Oliveira Mangabeira, Ricardo Pedro Moreira Dias, Francisco M. Couto, Carlos Priminho Pirovani

**Affiliations:** 1 Department of Biological Science, State University of Santa Cruz, Ilhéus, Bahia Brazil; 2 Cocoa Research Center, Ceplac/Cepec, Itabuna, BA Brazil; 3 BioISI: Biosystems & Integrative Sciences Institute, Faculdade de Ciências, Universidade de Lisboa, Lisbon, Portugal; 4 LaSIGE, Faculdade de Ciências, Universidade de Lisboa, Lisbon, Portugal

**Keywords:** *Theobroma cacao*, Leaf, Microbiome, Diversity

## Abstract

**Background:**

*Theobroma cacao* L. (cacao) is a perennial tropical tree, endemic to rainforests of the Amazon Basin. Large populations of bacteria live on leaf surfaces and these phylloplane microorganisms can have important effects on plant health. In recent years, the advent of high-throughput sequencing techniques has greatly facilitated studies of the phylloplane microbiome. In this study, we characterized the bacterial microbiome of the phylloplane of the catongo genotype (susceptible to witch’s broom) and CCN51 (resistant). Bacterial microbiome was determined by sequencing the V3-V4 region of the bacterial 16S rRNA gene.

**Results:**

After the pre-processing, a total of 1.7 million reads were considered. In total, 106 genera of bacteria were characterized. Proteobacteria was the predominant phylum in both genotypes. The exclusive genera of Catongo showed activity in the protection against UV radiation and in the transport of substrates. CCN51 presented genus that act in the biological control and inhibition in several taxonomic groups. Genotype CCN51 presented greater diversity of microorganisms in comparison to the Catongo genotype and the total community was different between both. Scanning electron microscopy analysis of leaves revealed that on the phylloplane, many bacterial occur in large aggregates in several regions of the surface and isolated nearby to the stomata.

**Conclusions:**

We describe for the first time the phylloplane bacterial communities of *T. cacao*. The Genotype CCN51, resistant to the witch’s broom, has a greater diversity of bacterial microbioma in comparison to Catongo and a greater amount of exclusive microorganisms in the phylloplane with antagonistic action against phytopathogens.

**Electronic supplementary material:**

The online version of this article (10.1186/s12866-018-1339-9) contains supplementary material, which is available to authorized users.

## Background

The phylloplane alone represents the largest biological surface on Earth, outnumbering the cells of the plants themselves [1, 2]. The microorganisms that live in this region multiply and occupy newly formed niches while the leaves are expanding [2, 3]. They are influenced by sunlight and the plants metabolism that have nutrients, including carbohydrates, organic acids and amino acids [4, 5]. Furthermore, the cuticle reduces water evaporation as well as leaching the metabolites in the leaves, resulting in a favorable environment [6–8]. These surfaces are an open environment that receive migrants transferred by various mechanisms including rain, animals and deposition of aerial particles, which contributes to a large microbial diversity [2, 9].

The phylloplane microorganisms can be shared randomly among its neighbors, but their survival and presence is generally regulated by the plant [10]. Furthermore, a theory proposes a possible transfer of microorganisms through generations [11]. However, this whole microbiome can be affected by environmental factors, including radiation [12] and pollution [13], as well as biotic factors such as leaf age and the presence of other microorganisms [14].

The microbiome present in the phylloplane includes a diversity of bacteria, fungi, yeasts, algae and other microorganisms that have commensal, pathogenic and mutualistic interactions with the plant [2, 15, 16]. Bacteria are numerically dominant in the phylloplane environment, of which a large part are proteobacteria, actinobacteria and bacteroidetes [9, 15, 17–22]. This variation is observed in different species of plants that have characteristic communities of bacteria in the phylloplane, varying between genotypes [23, 24], as well as between species and taxonomic classifications [17].

The microbial activities in the leaves can significantly influence the plant health [25–27]. The nitrogen fixation in the phylloplane is the main mechanism for the addition of nitrogen in tropical humid ecosystems [28] and temperate forests [29]. Plants can still be affected by the production of growth hormones [27] and indirect protection against pathogens [3, 25, 30]. In this environment, the cuticle [8] and the trichomes [31, 32] are also considered defense components in the phylloplane that together with the microorganisms, constitute a complex region [33, 34].

Next-generation sequencing technology (NGS) had a great impact in the microbial genomics field [35, 36]. The method provides new insights into the non-cultivable microorganisms and the complex host-microbe interactions [19, 37]. In this approach, the metagenomics used a hypervariable region of the highly conserved 16S rRNA gene as a phylogenetic marker allowing the characterization of the diversity of organisms of the total microbiota found in a given habitat [9, 20, 21, 38–40].

*Theobroma cacao* L. is a plant native to the South American rainforest, belonging to the Malvaceae family [41]. It is considered that this plant has two large groups of origin according to their morphological and genetic characteristics and geographic location [42, 43]. The cocoa has great industrial importance since it is the raw material for chocolate [44]. Currently, the genotype CCN51 is the most commercialized clone in several countries due to its great productivity. In contrast, the Catongo genotype is used as a model of sustainability of the fungus *Moniliophthora perniciosa* [31, 45], which causes witches’ broom disease in cocoa trees.

The CCN51 genotype is resistant to the fungus *Moniliophthora perniciosa* [45], and its phylloplane has twice as greater index of short glandular secreting trichomes than the Catongo genotype. A total of 41 proteins from leaf water washes (LWW) of the CCN51 identified by mass spectrometry revealed 28 plant proteins and 13 bacterial proteins [31]. This variation of the short glandular secreting trichomes index between the two genotypes may interfere in the microbial community of the phylloplane.

In this study, we propose that two contrasting genotypes for resistance to witches’ broom have variations in the phylloplane microbiota. Therefore, using an independent culture approach, the total microbiome of the *T. cacao* phyloplane of the genotypes CCN51 and Catongo, were studied and characterized. We show that the differences in the index of glandular trichomes in the contrasting genotypes may affect the variety of bacterial microbioma symbiont of the phylloplane, and that the CCN51 genotype presents an exclusive genera with antagonistic potential against phytopathogens in relation to the catongo genotype, reinforcing its preference of agriculture for the cultivation and commercialization.

## Materials and methods

### Plant material and DNA extraction

A total of 300 plants of *Theobroma cacao* L. were cultivated in the greenhouse at CEPEC / CEPLAC (Cacao Research Center, Ilhéus-BA); 150 plants of the Catongo genotype and 150 of the CCN51 genotype. The plants were kept at room temperature and drip irrigation to avoid leaf washing. Seven pots of plants were randomly selected from each genotype to form the pool of the first biological sample, and another second group, also with seven pots of plants to form the pool of the second biological sample (Additional file 1: Figure S1). Therefore, the four biological samples (two from CCN51 and two from Catongo) underwent the extraction of the metagenomic DNA and analyzed in triplicates experimental (Additional file 2: Figure S2).

Young leaves were collected within 15 to 20 days after leaf primordium formation, and the metagenomic DNA extraction from the phylloplane was obtained through leaf water wash according to the method described by Shepherd [46]. Furthermore, other young leaves of the CCN51 and Catongo genotypes were collected with the purpose of observing the phylloplane topography and microbes using the Scanning Electron Microscope (SEM) Quanta 250 model (FEI Company).

For extraction of total DNA, each leaf was washed by immersion for 15 s in a beaker containing 100 ml of distilled water maintained at temperature 8 °C. The microbiota was obtained from the LWW by filtering through a 0.22 μm cellulose membrane to retain the microorganisms. Afterwards, the membrane was distributed in eppendorf tubes, flash-frozen in liquid nitrogen and freeze dried until complete elimination of water. 0.5 g of freeze-dried membrane was weighed and DNA was extracted using the PowerSoil® DNA Isolation Kit (MoBio Laboratories, USA) according to the manufacturer’s instructions. DNA quality was checked on 0.8% (*w*/*v*) agarose gel and concentration and purity measured using the Nanodrop (Thermo Scientific, USA).

### Library construction and sequencing

Bacterial 16S rRNA gene sequences of the V3-V4 hypervariable region were amplified by PCR using the (341*F*) forward and (805*R*) reverse primer [47]. PCR was performed in a final volume of 25 μL containing the following: 2 μL of template DNA, 12.5 μL of HiFi HotStart ReadyMixPCR Kit (Kapa Biosystems) and 5 μL of each oligonucleotide. Amplification was performed on the Mx 3005P apparatus (Agilent Technologies) under the following conditions: 95 °C for 3 min, followed by 25 cycles of 95 °C for 30 s, 55 °C for 30 s and 72 °C for 30 s, and a final elongation step at 72 °C for 5 min.

Amplicons from each biological replicate (3 amplifications for each of the four DNA extractions) were purified using the Agencourt® AMPure® XP system (Beckman Coulter, USA). The quality of the purified amplicons was evaluated in 1.5% agarose gel. A new PCR with Nextera XT Index Kit (FC-131–1002) with final volume of 50 μL was performed in order to add the barcodes, using dual indexing strategy with two 8-base indices. The new amplification was performed under the same conditions as the previous PCR, except for the number of cycles (8). After quantification of the 12 samples using the Kapa Library Quantification kit (Additional file 3: Figure S3), the libraries were sequenced on the Illumina MiSeq™ equipment using the V3 kit (MiSeq® Reagent - Illumina).

### Data analysis

Raw bacterial sequence reads were initially subjected to the following preprocessing steps and quality controls: (i) ≤ 100 nucleotides in length (not including sample barcodes) or more than 600 bp were not considered and (ii) reads were trimmed at the beginning of a poor quality region with 10 bp analyzed in FastQC [48] software with a Phred-score ≤ 20. In subsequent screenings, files were processed using MeFit [49], to identify the best possible overlap region, with the least number of mismatching bases and carry out the merger.

Files were demultiplexed and end chimeras removed using the Quantitative Insights into Microbial Ecology (QIIME) [50] software package and operational taxonomic units (OTUs) were assigned by clustering the sequences with a threshold of 99% identity against the Greengen database version 13.8 16S rRNA [51]. OTUs, were assigned to “chloroplasts” and “mitochondria” before it was rarefied and served as input for alpha and beta diversity analysis, were filtered. The Qiime package generated rarefaction curves (richness of population analysis) and the calculation of the population diversity analysis estimator Chao1, as also, Alpha (within-sample richness) and beta diversity (between-sample dissimilarity) estimate. Using the GeanAIEx [52] software, the Principal Coordinates Analysis (PCoA) chart was plotted according to weighted UniFrac metrics (β-diversity). To test whether there is a significant difference in bacterial community composition among genotypes CCN51 and Catongo, we used the analysis of similarity (ANOSIM) with 999 permutations [53].

## Results

**Fig. 1:**
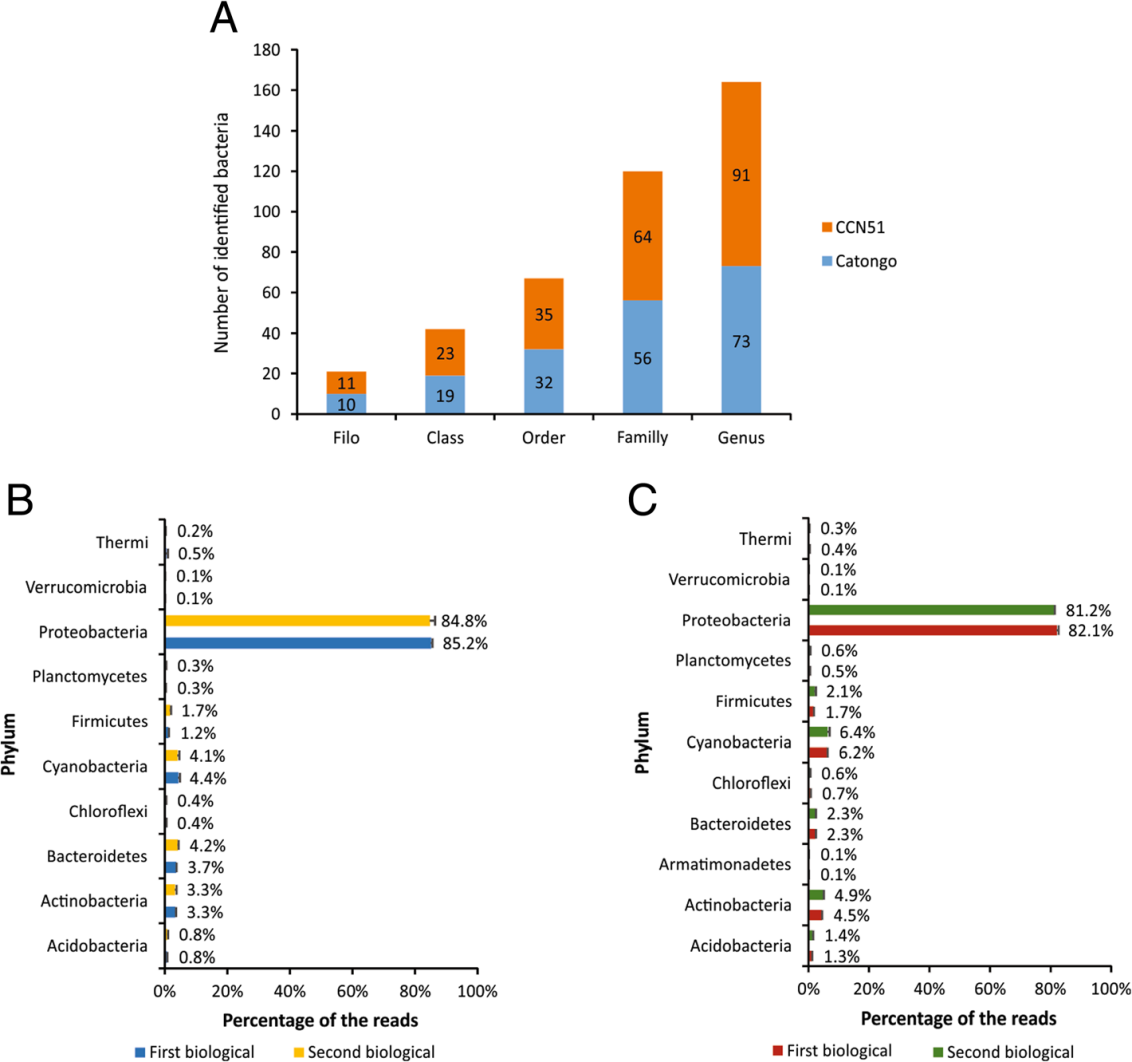
Distribution of identified bacteria in leaf water washes of *Theobroma cacao*. **a** Distribution of the number of bacteria identified. Distribution of frequency of reads identified according to phylum: (**b**) Catongo genotype and (**c**) CCN51 genotype. Error bars indicate the standard deviation between the frequencies of three experimental samples of each biological sample

After pre-processing, filtering and rarefaction, the sequencing produced a total of 1.7 million reads of the V3-V4 variable region of the 16S rRNA from leaf water washes of the two contrasting cacao genotypes for resistance of witches’ broom disease, caused by *M. perniciosa* fungus. The average number of reads per sample was 95.398, ranging from 42.068 to 346.420. The identified bacteria were classified according to phylum, class, order, family, and genus (Additional file 4: Table S1). A total of 10 phyla and 73 genera were identified in the Catongo genotype and a total of 11 phyla and 91 genera in the CCN51 genotype (Fig. 1a). At the phylum level, proteobacteria is the most abundant phylum in the two genotypes, followed by cyanobacteria also in both genotypes and by bacteroidetes in the genotype Catongo (Fig. 1b) and actinobacteria in the CCN51 genotype (Fig. 1c).

**Fig. 2:**
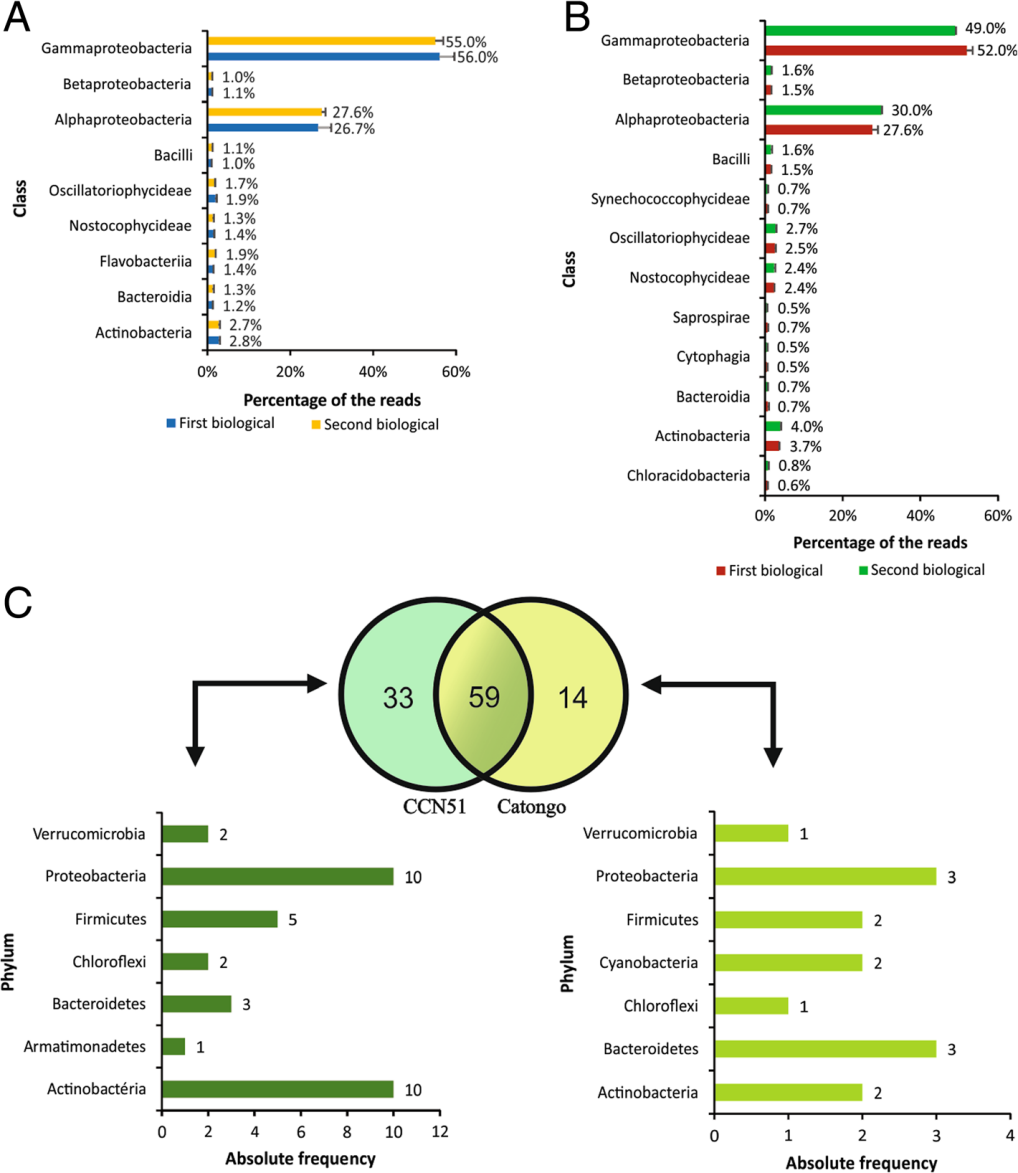
Distribution of the frequency of identified bacteria according to class in leaf water washes from contrasting cacao genotypes for *M. perniciosa* resistance. **a** susceptible Catongo genotype. **b** resistant CCN51 genotype. Only classes that represent ≥1% of the total population in at least one sample, are included. Error bars indicate the standard deviation between the frequencies of three experimental samples of each biological sample. **c** Comparison of the number of identified bacterial phylum in the phylloplane between two genotypes

The three dominant bacterial taxonomic classes in the Catongo and CCN51 genotypes were Gammaproteobacteria, Alphaproteobacteria and Actinobacteria (Fig. 2a and b). Among the orders identified (Fig. 1a), oceanospirillales, rickettsiales and enterobacteriales, were the three most abundant orders in both genotypes, 45.9, 21.6 and 9.6% for Catongo and, 43.0, 20.1 and 6.6% for CCN51, respectively.

At the family level, oceanospirillales (44.3%) is the most abundant order in the CCN51 genotype, followed by rickettsiales (20.7%) and enterobacteriales (6.8%). Rickettsiales (42.0%) is the most abundant order followed by enterobacteriales (18.7%) and sphingomonadales (4.9%), in the Catongo genotype. Comparison at genus level was carried out to reveal bacteria commonly or specifically identified in both genotypes, (Fig. 2c). Genotype CCN51 presented 33 genera of bacteria exclusive to its phylloplane in comparison to Catongo. The *Stenotrophomonas* genus was prevalent with 13.5% exclusively found in the CCN51 genotype, whereas the genus *Sphingomonas* prevailed with 75.3%, in the Catongo genotype (Additional file 5: Table S2).

**Fig. 3:**
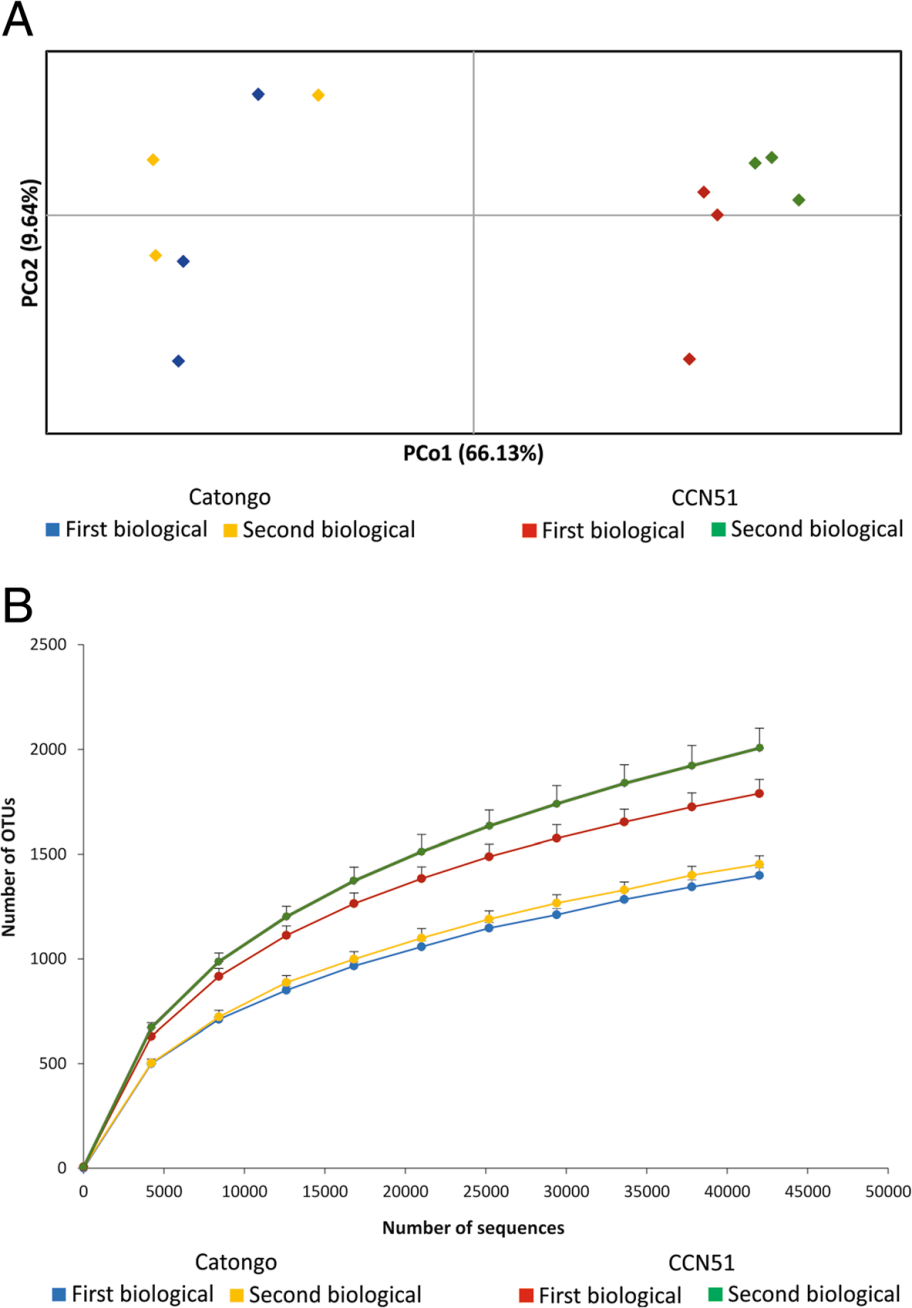
PCoA plot and rarefaction curves determined for all 12 samples of microorganisms from phylloplane of the two contrasting cacao genotypes for *M. perniciosa* resistance. **a** Principal Coordinates Analysis (PCoA) between bacterial communities. **b** Rarefaction curves demonstrating species richness (Chao1) and diversity (PD entire tree)

In terms of bacterial diversity, rarefaction curves generated from the library reads (42.000 reads per sample) based on a cutoff 99% sequence identity showed an asymptote for both genotypes, which tended to stabilize indicating sufficient sampling to capture most OTUs within communities (Fig. 3b). The graphic analysis showed the differences in biodiversity, because the genotype CCN51 represents the curves of the upper part of the figure, revealing that microbial communities from genotype CCN51 were more diverse than those from microbial communities from the genotype Catongo. The PCoA analysis based on weighted UniFrac metrics showed that the bacterial community were clustered per genotype (Fig. 3a), indicating the distinct bacterial diversity between CCN51 and Catongo. The ANOSIM results also showed that there is a significant difference in bacterial composition between genotypes CCN51 and Catongo (Global *R* = 0.996, *P* < 0.05).

**Fig. 4:**
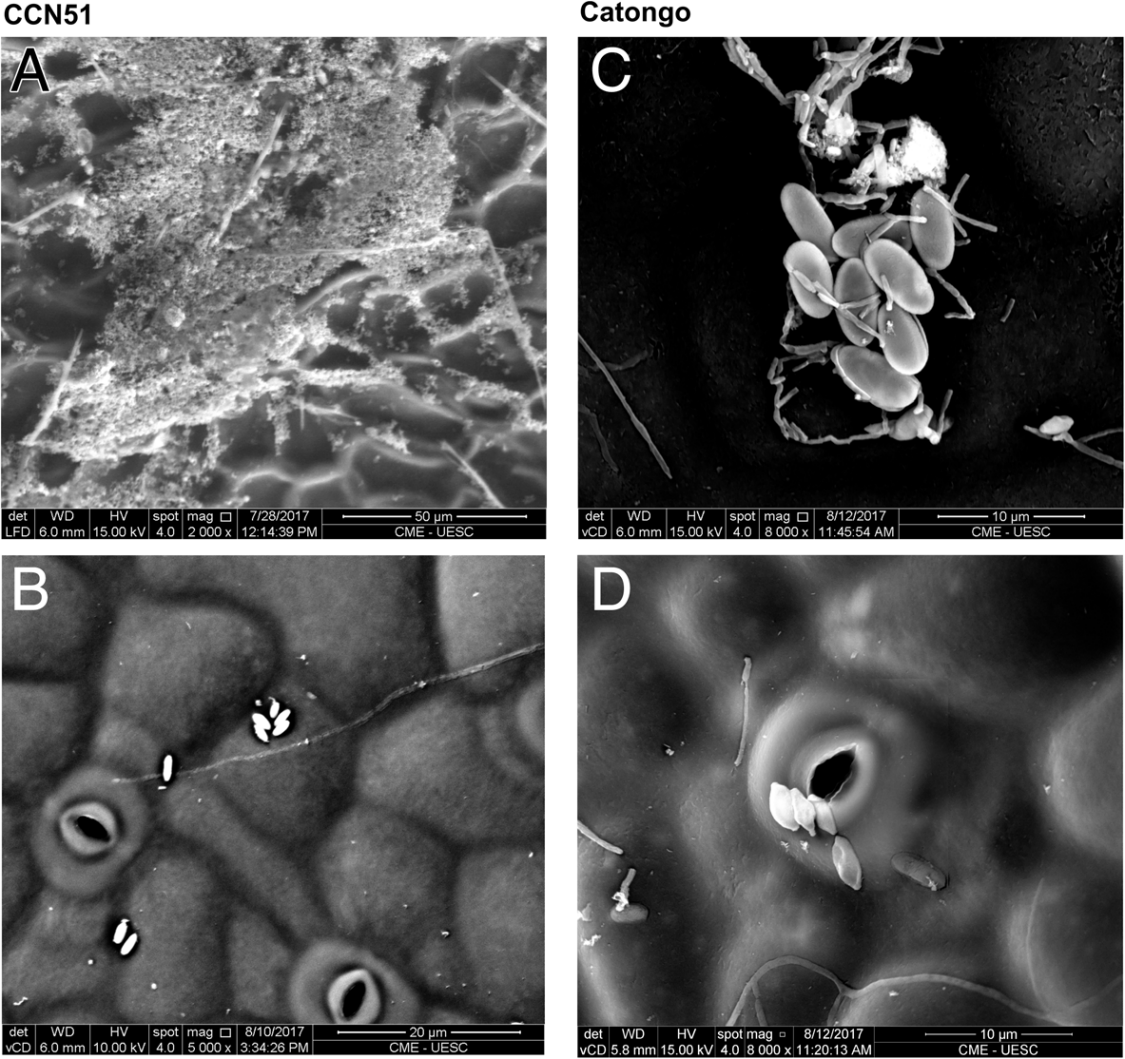
Scanning electron microscopy analysis. **a** Adaxial surface of the CCN51 genotype. **b** Abaxial surface of the CCN51 genotype. **c** Adaxial surface of the Catongo genotype. **d** Abaxial surface of the Catongo genotype

Electron microscopy analysis of the leaves revealed that in the phylloplane many epiphytes occur in large bacterial aggregates, and fungi (Fig. 4). The field images provides spatial view of microbiome locations. Isolated bacterial cells have also been observed and some mixed aggregates can be found. The bacteria were visualized next to stomates, cell junctions and mainly in the foliar veins.

## Discussion

Cocoa is a source of raw material for chocolate production and is cultivated in tropical and subtropical regions around the world [44]. Great losses in cacao production happen due to fungal diseases, such as witches’ broom and frosty pod, caused by *M. perniciosa* and *M. roreri* [54]*,* respectively. These pathogens are hemibiobrophyc and start the infection process with spores deposited on the phylloplane from young cacao tissues (leaf and fruits). There is no effective chemical control after the invasion of the apoplast and onset of the parasitic phase of the disease [55, 56]. The mechanisms of spore germination and pre-infection processes in contrasting resistance cacao genotypes has been analyzed [57]. Furthermore, the topography of the epicuticular wax layer [58], the short glandular secreting trichomes and the importance of water-soluble components of the phylloplane [31] have also been analyzed. *M. perniciosa* tends to have a relatively short epiphytic phase [31, 59] and requires few or no exogenous nutrients in this phase [60]. Thus, if biocontrol occurs at the epiphytic phase, antagonists that act as antibiotics (rather than competition) should be the most effective [61]. In this work, we analyze the composition and bacterial diversity of the phylloplane of CCN51 and Catongo cacao genotypes, resistant and susceptible to witches’ broom respectively. DNA extracted from microorganisms recovered by filtration from leaf water washes was analyzed by metagenomic approach based on the sequencing of the v3-v4 hypervariable region from the 16S rRNA.

Microbiome communities contained abundance of genera within the proteobacteria and cyanobacteria phylum, with prevalence of *Candidatus Portiera* in both genotypes (Additional file 4: Table S1). This genus presented a single species *Candidatus Portiera aleyrodidarum* sp. which provides amino acids and carotenoids [62] to its host *Bemisia tabaci* [63]. In the phylloplane, this microorganism may be acting symbiotically with *T. cacao*, participating in the organic metabolism with the contribution of amino acids tryptophan and also participating in the photosynthesis providing carotenoids [62, 64]. Tryptophan may be involved in the cellular elongation of young leaves of *T. cacao*, as this amino acid is a precursor of indolylacetic acid, a growth hormone [65, 66].

Gammaproteobacteria is dominant in the taxonomic composition at class level of phylloplane communities of *T. cacao* (Fig. 2). It differs from tropical and temperate community structures already described. Phylloplane communities in Canadian forests were dominated by Alphaproteobacteria (68%) [40], contrasting with 27% in Malaysia [21] and 22.8% in Panama [67] in tropical trees. However, percentages of Alphaproteobacteria in trees of tropical climates were similar to percentages found in *T. cocoa*. Some studies report that phylloplane bacteria vary among plants of different developmental stages and genotypes [10, 29]. The bacterial diversity in the phylloplane appears to be as high as that presented in roots or in the human gut [10]. Others describe that microbial diversity and plant species may change according to the environment, climate and geography [22, 26, 40, 68], revealing patterns of change in the phylloplane microbial communities of each species across geographically separated ecosystems. The large diversity of microbiota at the phylloplane may also influence plant evolution, as described by the hologenome theory. Both host and symbiont genomes can be transmitted from one generation to the next [11].

*Sphingomonas* (75.3%), the predominant genus among the exclusive ones in Catongo, has a pigmentation which confers protection against UV radiation to the phylloplane [2], also assists in transportation of substrates (e.g. sugars, vitamins, siderophore) [22] and acts as regulator of stress-related responses, such as PhyR and EcfG [26]. Several members of the genus *Sphingomonas* isolated from plants (*Arabidopsis thaliana*, *Acacia caven*, *Oryza sativa* and *Nicotiana tabacum*) conferred protection in *A. thaliana* against *Pseudomonas syringae* and *Xanthomonas campestris*, reducing disease symptoms or diminishing pathogen growth in the phylloplane [69]. *Sphingomonas melonis* and *Methylobacterium extorquens* demonstrated a profound impact on the transcriptome of the plant *Arabidopsis thaliana*, researchers found that the expression of nearly 400 genes may be involved in the plant defense responses [70]. Nonetheless, *Stenotrophomonas* is the predominant genus (13.5%) among the exclusive ones in CCN51 and it was reported as being characteristic from plant leaves (*Chlorophytum comosum*, *Olea europaea* and *Dracaena draco*), that grow in cold temperate climates [71]. The regulation system of pathogenicity factors (Rpf) and diffusible signal factor (DSF), are also conserved in this type of genus [72]. Other predominant genera among the exclusive ones in CCN51, were *Lysobacter* (5.89%) and *Paenibacillus* (3.13%). *Lysobacter* spp. has been shown to be important as biological control agents, producing both antibiotics and enzymes capable of degrading the cell walls from host fungi in *Cucumis sativus* and *Solanum lycopersicum* [73, 74]. In contrast, a species of the genus *Paenibacillus* (*P. peoriae*), demonstrated a broad inhibition spectrum in several taxonomic groups of bacteria and fungi [75].

Rarefaction analyses (Fig. 3b) and PCoA analysis show that the total bacterial diversity in the genotype CCN51 phylloplane was larger in comparison to the Catongo genotype and the bacterial community is clustered as per the genotype type (Fig. 3a). According to the rarefaction curve, the CCN51 genotype of *T. cacao* showed higher OTUs than the Catongo genotype. The rarefaction curves trends to plateau suggests that a good coverage of the entire community of the phylloplane was achieved. The difference in the curve between the biological samples of the CCN51 genotype can be explained by the interval of 15 days between the collection of the first and second biological samples. The highest index of short glandular secreting trichomes that occur in the witch’s broom-resistant CCN51 genotype compared to the susceptible Catongo [31], may affect the amount and variety of proteins and metabolites released into the phylloplane [2]. We believe that it might be the cause of the qualitative and quantitative differences in the microbial community of the two genotypes shown in the results. Furthermore, these phylloplane variations, due to plant metabolites and of the microbial community, together with variations in the topography of the phylosphere between genotypes (Fig. 4), strongly suggests that they may contribute to the differences in resistance to disease occurring between the CCN51 and Catongo genotypes [31].

Bacterial communities presented distinct colonization patterns in the *T. cacao* phylloplane (Fig. 4). Some studies have described that penetration of the germinal tube of the fungus *M. perniciosa* [76, 77], and colonization of other microorganisms, can occur at the base of the glandular trichoma, junctions of the cells, sites of lesions, stomata, and in the veins [2, 78], and may undergo changes at different seasons and age of the leaf. The diversity is lower during hot and dry months and higher during rainy and cold seasons [79]. In young leaves, communities are made up of a greater number of microorganisms relative to mature and senescent leaves, as well as at different seasons [79, 80]. The formation of aggregates by bacteria may constitute between 30 and 80% of the total bacterial population in certain species of plants [81].

In the rice phylloplane, the microbiome presented greater diversity in cultivated and controlled plants in pots than those cultivated in the open field [82]. Bacterial community composition in phylloplane of *Deschampsia antarctica* at different locations in open fields, revealed significant differences [83]. The phylloplane and its microbial communities are interrelated [84] and can provide a structural and functional model microenvironment [3, 85] to understand plant-pathogen interactions and thus to select more resistant plants, which will contribute to the continuity of food production.

## Conclusions

In this study, to the best of our knowledge, we describe for the first time the phylloplane bacterial communities of *T. cacao*. In addition, we performed the first evaluation of hosts identity and an analysis of diversity in two contrasting genotypes for witch’s broom resistance. Proteobacteria is the most abundant phylum in the two genotypes, with prevalence of *Candidatus Portiera* in both. Genotype CCN51, resistant to the witch’s broom, has a greater diversity of bacterial microbioma in comparison to Catongo and also greater amount of exclusive microorganisms in the phylloplane with antagonistic action against phytopathogens. The bacterial diversity among phylloplane populations are distinct between the genotypes according the PCoA analysis and validated by statistics ANOSIM that showed a significant difference in bacterial composition between genotypes CCN51 and Catongo. The study revealed the importance of epiphytic microbiome and may be a highly valuable tool in the process of biological control. The findings will be of great value for improving the understanding of the defense and interaction mechanisms that occur in the phylloplane.

## Additional files

Additional file 1: **Figure S1.** Distribution of plants in the greenhouse. (A) Selected plants: green (first biological), red (second biological). (B) Panoramic photo of plants. (DOCX 2745 kb)

Additional file 2: **Figure S2.** Extraction of the metagenomic DNA in triplicates experimental. (A) First biological sample - CCN51. (B) First biological sample - Catongo. (C) Second biological sample - CCN51 and (D) Second biological sample - Catongo. (DOCX 151 kb)

Additional file 3: **Figure S3.** Quantification of libraries. (A) Electrophoresis on 1% (w / v) agarose gel with the six standards, 12 libraries (quantified in triplicates) and three negative controls – a, b and c: first biological sample - CCN51; d, e and f: first biological sample - Catongo; g, h and i: second biological sample - CCN51; j, k and l: second biological sample - Catongo; NC: negative control. (B) Dissociation curve – a: libraries; b: negative control. (DOCX 307 kb)

Additional file 4: **Table S1.** Bacteria identified and classified according to phylum, class, order, family, and genus for in the genotypes CCN51 and Catongo, with a threshold of 99% identity against the Greengen database version 13.8 16S rRNA. (DOCX 19 kb)

Additional file 5: **Table S2.** Bacterial genera exclusive to the phylloplane of the genotypes CCN51 and Catongo. (DOCX 17 kb)

## Abbreviations

ANOSIM: Analysis of similarity
DSF: Diffusible signal factor
LWW: Leaf water washes
NGS: Next-generation sequencing technology
OTUs: Operational taxonomic units
PCoA: Principal coordinates analysis
PCR: Polymerase chair reaction
QIIME: Quantitative Insights Into Microbial Ecology
Rpf: System of regulation of pathogenicity factors
SEM: Scanning Electron Microscope
