# Supplementary material for: Witches’ broom resistant genotype CCN51 shows greater diversity of symbiont bacteria in its phylloplane than susceptible genotype catongo
Source: BMC Microbiol. 2018 Nov 23;18:194. doi: 10.1186/s12866-018-1339-9 (PMC6251189; doi:10.1186/s12866-018-1339-9)
Supplement: Supplementary file 1 — Figure S1. Distribution of plants in the greenhouse. (A) Selected plants: green (first biological), red (second biological). (B) Panoramic photo of plants. (DOCX 2745 kb) [file 12866_2018_1339_MOESM1_ESM.docx]

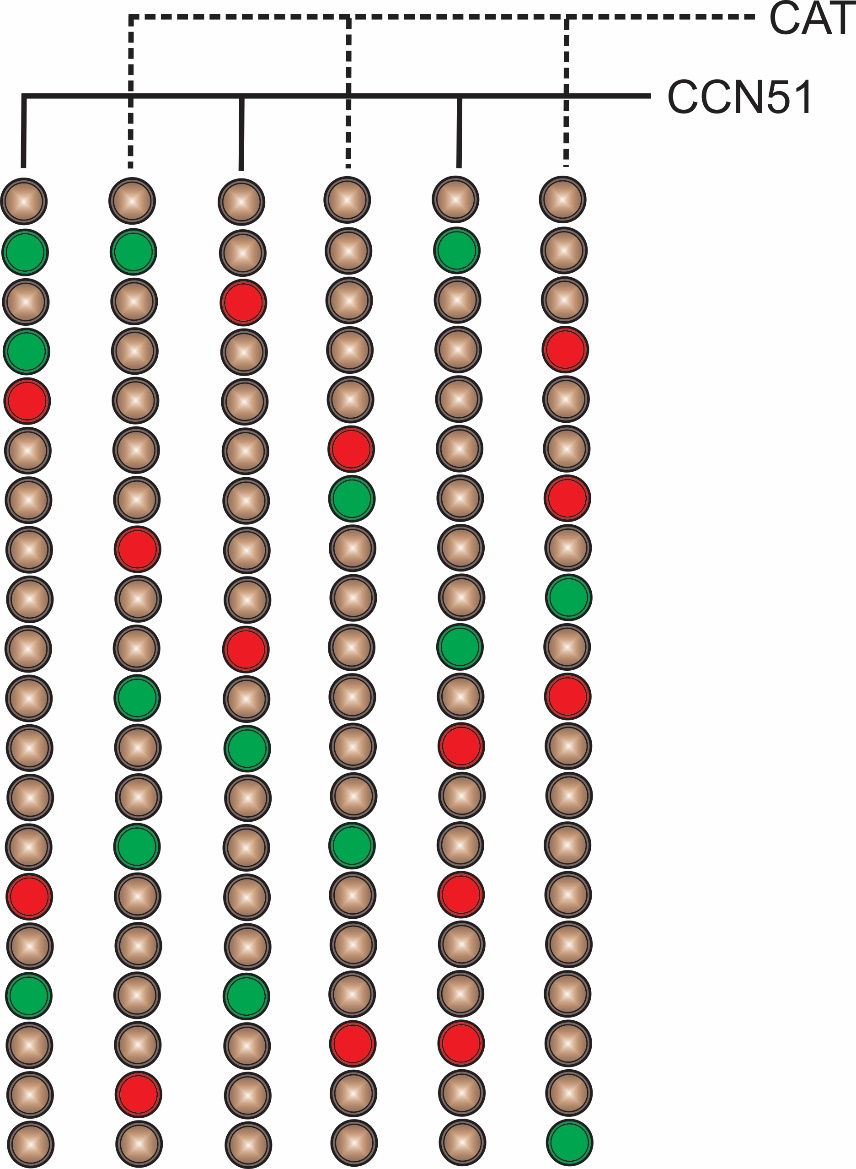


**A**

**B**


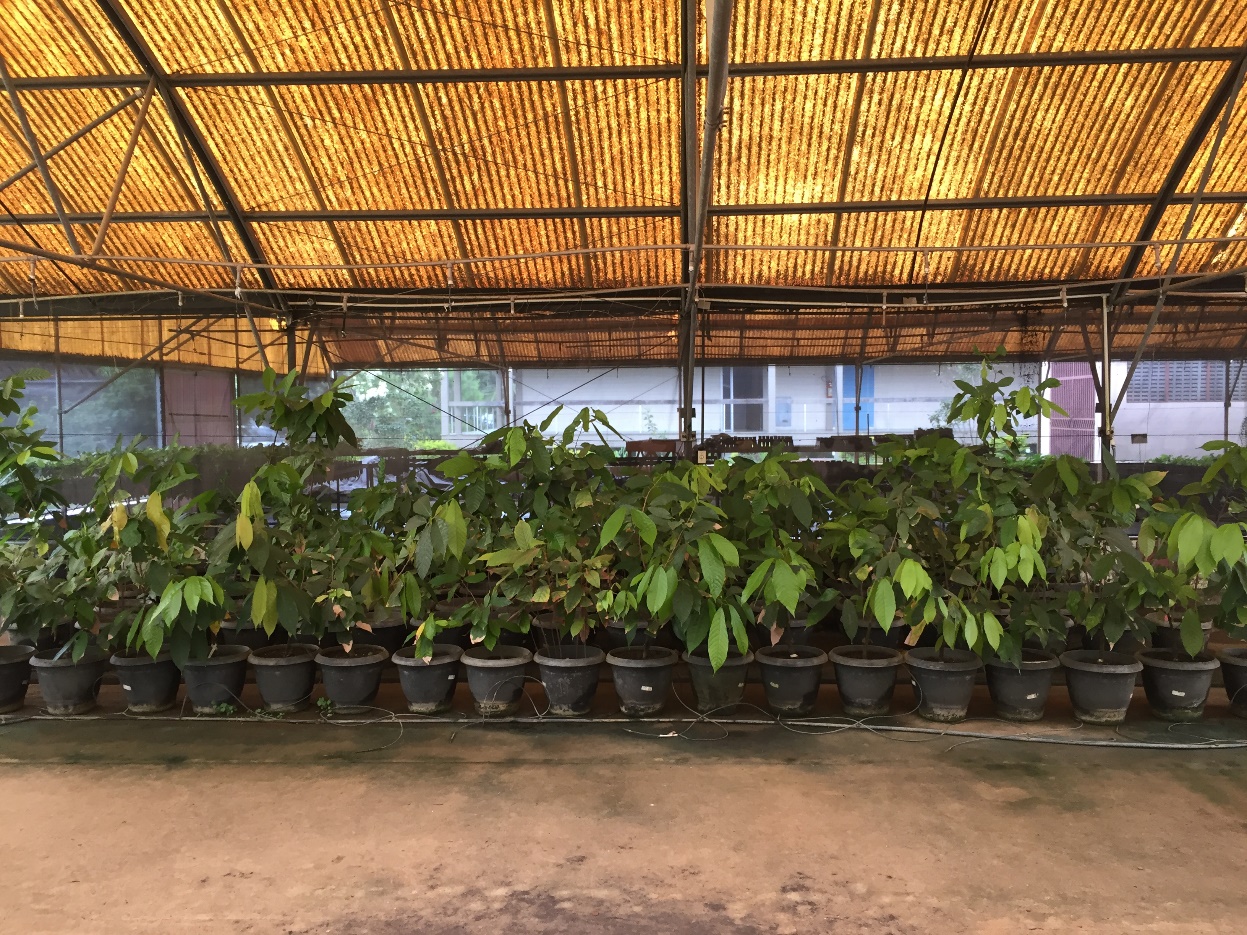


**Figure S1.** Distribution of plants in the greenhouse. (A) Selected plants: green (first biological), red (second biological). (B) Panoramic photo of plants.
