# Supplementary material for: Witches’ broom resistant genotype CCN51 shows greater diversity of symbiont bacteria in its phylloplane than susceptible genotype catongo
Source: BMC Microbiol. 2018 Nov 23;18:194. doi: 10.1186/s12866-018-1339-9 (PMC6251189; doi:10.1186/s12866-018-1339-9)
Supplement: Supplementary file 2 — Figure S2. Extraction of the metagenomic DNA in triplicates experimental. (A) First biological sample - CCN51. (B) First biological sample - Catongo. (C) Second biological sample - CCN51 and (D) Second biological sample - Catongo. (DOCX 151 kb) [file 12866_2018_1339_MOESM2_ESM.docx]

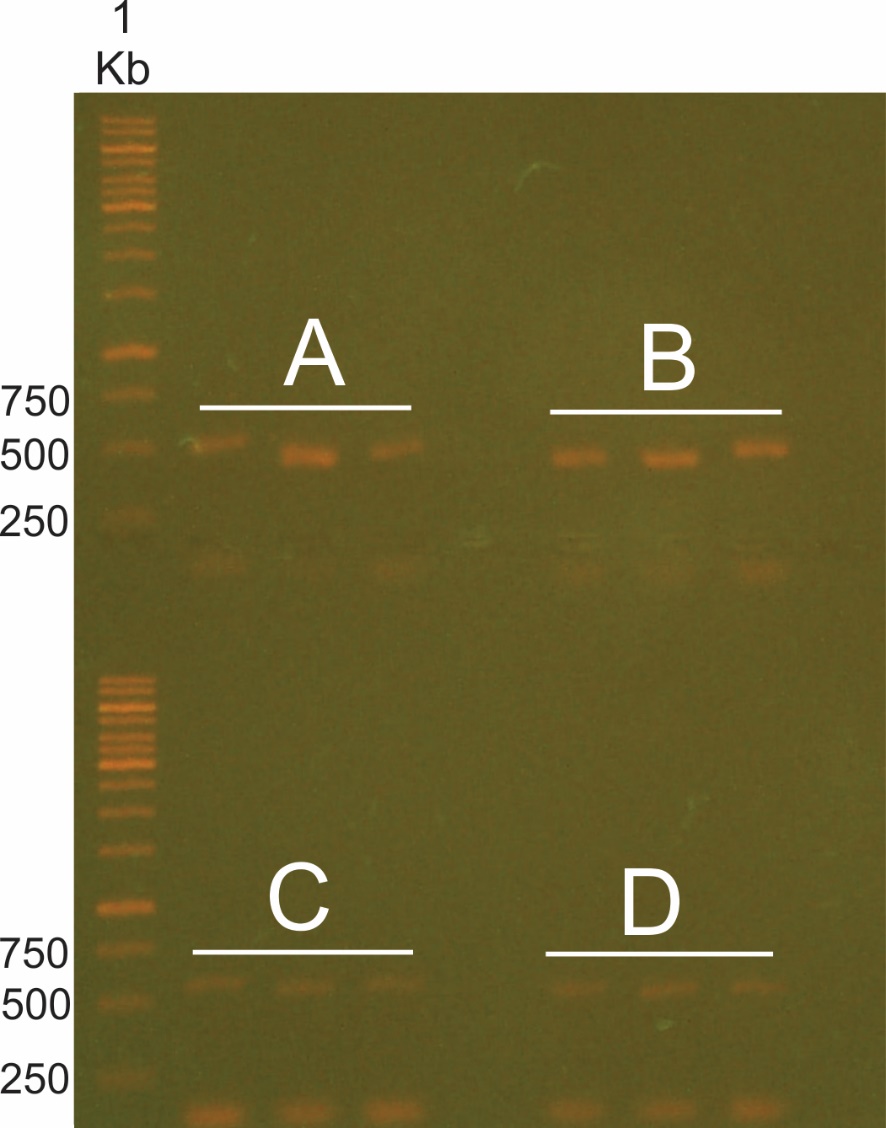


**Figure S2.** Extraction of the metagenomic DNA in triplicates experimental. (A) First biological sample - CCN51. (B) First biological sample - Catongo. (C) Second biological sample - CCN51 and (D) Second biological sample - Catongo.
