# Supplementary material for: Witches’ broom resistant genotype CCN51 shows greater diversity of symbiont bacteria in its phylloplane than susceptible genotype catongo
Source: BMC Microbiol. 2018 Nov 23;18:194. doi: 10.1186/s12866-018-1339-9 (PMC6251189; doi:10.1186/s12866-018-1339-9)
Supplement: Supplementary file 3 — Figure S3. Quantification of libraries. (A) Electrophoresis on 1% (w / v) agarose gel with the six standards, 12 libraries (quantified in triplicates) and three negative controls – a, b and c: first biological sample - CCN51; d, e and f: first biological sample - Catongo; g, h and i: second biological sample - CCN51; j, k and l: second biological sample - Catongo; NC: negative control. (B) Dissociation curve – a: libraries; b: negative control. (DOCX 307 kb) [file 12866_2018_1339_MOESM3_ESM.docx]

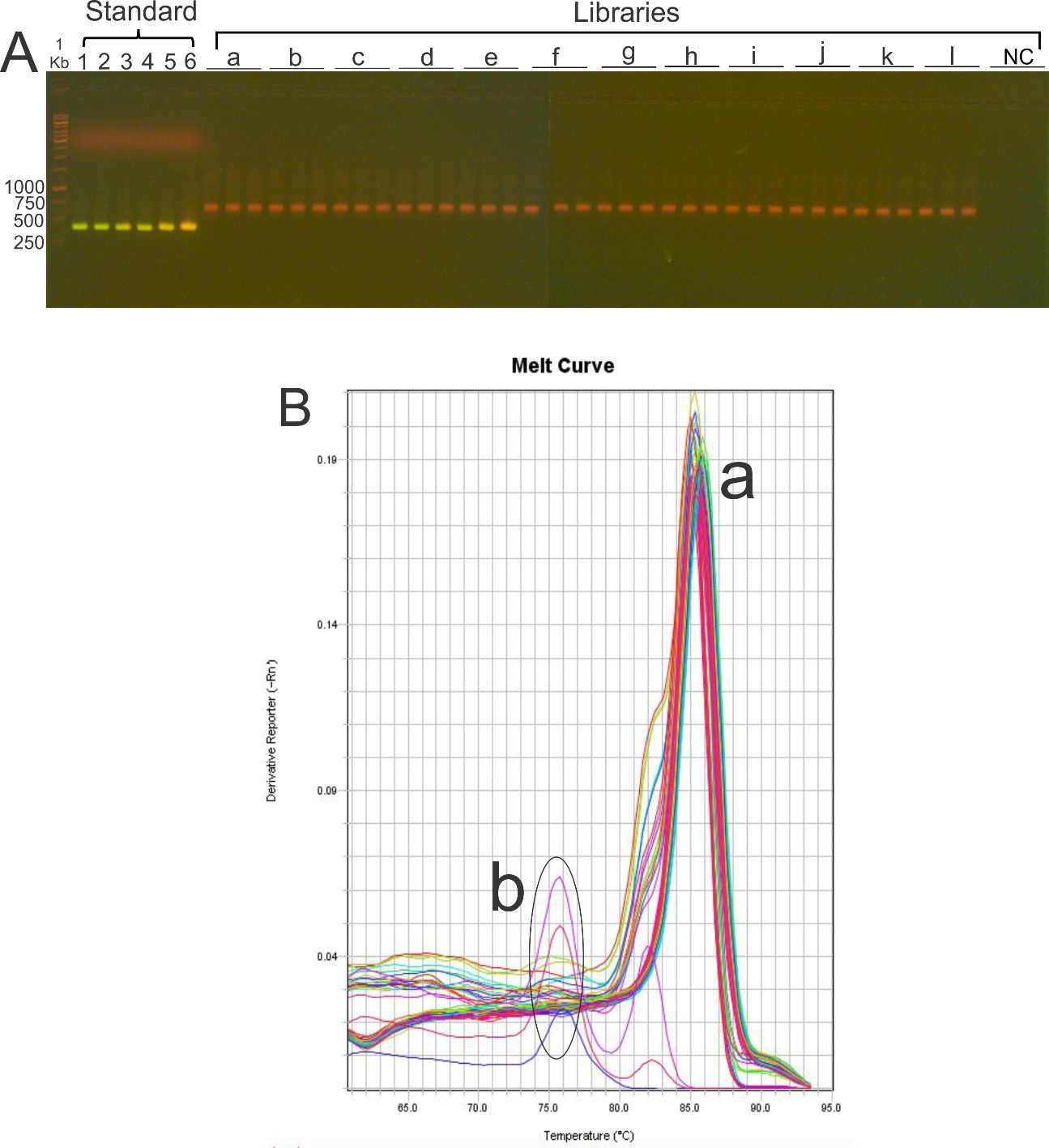


**Figure S3.** Quantification of libraries. (A) Electrophoresis on 1% (w / v) agarose gel with the six standards, 12 libraries (quantified in triplicates) and three negative controls – a, b and c: first biological sample - CCN51; d, e and f: first biological sample - Catongo; g, h and i: second biological sample - CCN51; j, k and l: second biological sample - Catongo; NC: negative control. (B) Dissociation curve – a: libraries; b: negative control.
