# Supplementary material for: Witches’ broom resistant genotype CCN51 shows greater diversity of symbiont bacteria in its phylloplane than susceptible genotype catongo
Source: BMC Microbiol. 2018 Nov 23;18:194. doi: 10.1186/s12866-018-1339-9 (PMC6251189; doi:10.1186/s12866-018-1339-9)
Supplement: Supplementary file 4 — Table S1. Bacteria identified and classified according to phylum, class, order, family, and genus for in the genotypes CCN51 and Catongo, with a threshold of 99% identity against the Greengen database version 13.8 16S rRNA. (DOCX 19 kb) [file 12866_2018_1339_MOESM4_ESM.docx]

**Table S1.** Bacteria identified and classified according to phylum (p), class (c), order (o), family (f), and genus (g) for in the genotypes CCN51 and Catongo, with a threshold of 99% identity against the Greengen database version 13.8 16S rRNA.

| **CCN51** | | |
| --- | --- | --- |
| **Nº** | **Identification** | **Percentage** |
| 1 | p__Acidobacteria;c__Solibacteres;o__Solibacterales;f__Solibacteraceae;g__Candidatus Solibacter | 0.09 |
| 2 | p__Actinobacteria;c__Actinobacteria;o__Actinomycetales;f__Actinomycetaceae;g__Actinobaculum | 0.03 |
| 3 | p__Actinobacteria;c__Actinobacteria;o__Actinomycetales;f__Brevibacteriaceae;g__Brevibacterium | 0.03 |
| 4 | p__Actinobacteria;c__Actinobacteria;o__Actinomycetales;f__Corynebacteriaceae;g__Corynebacterium | 0.05 |
| 5 | p__Actinobacteria;c__Actinobacteria;o__Actinomycetales;f__Dermabacteraceae;g__Brachybacterium | 0.17 |
| 6 | p__Actinobacteria;c__Actinobacteria;o__Actinomycetales;f__Kineosporiaceae;g__Kineococcus | 0.01 |
| 7 | p__Actinobacteria;c__Actinobacteria;o__Actinomycetales;f__Microbacteriaceae;g__Curtobacterium | 0.04 |
| 8 | p__Actinobacteria;c__Actinobacteria;o__Actinomycetales;f__Microbacteriaceae;g__Leucobacter | 0.03 |
| 9 | p__Actinobacteria;c__Actinobacteria;o__Actinomycetales;f__Microbacteriaceae;g__Microbacterium | 0.03 |
| 10 | p__Actinobacteria;c__Actinobacteria;o__Actinomycetales;f__Micrococcaceae;g__Arthrobacter | 0.03 |
| 11 | p__Actinobacteria;c__Actinobacteria;o__Actinomycetales;f__Micrococcaceae;g__Kocuria | 0.08 |
| 12 | p__Actinobacteria;c__Actinobacteria;o__Actinomycetales;f__Micrococcaceae;g__Micrococcus | 0.04 |
| 13 | p__Actinobacteria;c__Actinobacteria;o__Actinomycetales;f__Micrococcaceae;g__Nesterenkonia | 0.40 |
| 14 | p__Actinobacteria;c__Actinobacteria;o__Actinomycetales;f__Micromonosporaceae;g__Actinoplanes | 0.03 |
| 15 | p__Actinobacteria;c__Actinobacteria;o__Actinomycetales;f__Micromonosporaceae;g__Pilimelia | 0.03 |
| 16 | p__Actinobacteria;c__Actinobacteria;o__Actinomycetales;f__Mycobacteriaceae;g__Mycobacterium | 0.05 |
| 17 | p__Actinobacteria;c__Actinobacteria;o__Actinomycetales;f__Nocardiaceae;g__Nocardia | 0.03 |
| 18 | p__Actinobacteria;c__Actinobacteria;o__Actinomycetales;f__Nocardiaceae;g__Rhodococcus | 0.03 |
| 19 | p__Actinobacteria;c__Actinobacteria;o__Actinomycetales;f__Nocardioidaceae;g__Kribbella | 0.02 |
| 20 | p__Actinobacteria;c__Actinobacteria;o__Actinomycetales;f__Nocardioidaceae;g__Nocardioides | 0.04 |
| 21 | p__Actinobacteria;c__Actinobacteria;o__Actinomycetales;f__Promicromonosporaceae;g__Xylanimicrobium | 0.02 |
| 22 | p__Actinobacteria;c__Actinobacteria;o__Actinomycetales;f__Propionibacteriaceae;g__Microlunatus | 0.03 |
| 23 | p__Actinobacteria;c__Actinobacteria;o__Actinomycetales;f__Propionibacteriaceae;g__Propionibacterium | 0.69 |
| 24 | p__Actinobacteria;c__Actinobacteria;o__Actinomycetales;f__Pseudonocardiaceae;g__Actinomycetospora | 0.44 |
| 25 | p__Actinobacteria;c__Actinobacteria;o__Actinomycetales;f__Pseudonocardiaceae;g__Pseudonocardia | 0.60 |
| 26 | p__Actinobacteria;c__Actinobacteria;o__Actinomycetales;f__Streptomycetaceae;g__Streptomyces | 0.03 |
| 27 | p__Actinobacteria;c__Actinobacteria;o__Actinomycetales;f__Thermomonosporaceae;g__Actinomadura | 0.02 |
| 28 | p__Actinobacteria;c__Rubrobacteria;o__Rubrobacterales;f__Rubrobacteraceae;g__Rubrobacter | 0.44 |
| 29 | p__Armatimonadetes;c__[Fimbriimonadia];o__[Fimbriimonadales];f__[Fimbriimonadaceae];g__Fimbriimonas | 0.04 |
| 30 | p__Bacteroidetes;c__Bacteroidia;o__Bacteroidales;f__Porphyromonadaceae;g__Dysgonomonas | 0.88 |
| 31 | p__Bacteroidetes;c__Cytophagia;o__Cytophagales;f__Cytophagaceae;g__Adhaeribacter | 0.04 |
| 32 | p__Bacteroidetes;c__Cytophagia;o__Cytophagales;f__Cytophagaceae;g__Hymenobacter | 0.24 |
| 33 | p__Bacteroidetes;c__Cytophagia;o__Cytophagales;f__Cytophagaceae;g__Rhodocytophaga | 0.03 |
| 34 | p__Bacteroidetes;c__Cytophagia;o__Cytophagales;f__Cytophagaceae;g__Spirosoma | 0.05 |
| 35 | p__Bacteroidetes;c__Cytophagia;o__Cytophagales;f__Cytophagaceae;g__Sporocytophaga | 0.03 |
| 36 | p__Bacteroidetes;c__Flavobacteriia;o__Flavobacteriales;f__Flavobacteriaceae;g__Flavobacterium | 0.03 |
| 37 | p__Bacteroidetes;c__[Rhodothermi];o__[Rhodothermales];f__Rhodothermaceae;g__Rubricoccus | 0.03 |
| 38 | p__Bacteroidetes;c__[Saprospirae];o__[Saprospirales];f__Chitinophagaceae;g__Flavisolibacter | 0.41 |
| 39 | p__Chloroflexi;c__Chloroflexi;o__Chloroflexales;f__Chloroflexaceae;g__Chloronema | 0.01 |
| 40 | p__Chloroflexi;c__Chloroflexi;o__Chloroflexales;f__Oscillochloridaceae;g__Oscillochloris | 0.02 |
| 41 | p__Cyanobacteria;c__Nostocophycideae;o__Nostocales;f__Scytonemataceae;g__Brasilonema | 0.05 |
| 42 | p__Cyanobacteria;c__Nostocophycideae;o__Nostocales;f__Scytonemataceae;g__Scytonema | 0.27 |
| 43 | p__Cyanobacteria;c__Nostocophycideae;o__Stigonematales;f__Rivulariaceae;g__Calothrix | 0.72 |
| 44 | p__Cyanobacteria;c__Oscillatoriophycideae;o__Chroococcales;f__Xenococcaceae;g__Chroococcidiopsis | 0.19 |
| 45 | p__Cyanobacteria;c__Synechococcophycideae;o__Pseudanabaenales;f__Pseudanabaenaceae;g__Leptolyngbya | 0.48 |
| 46 | p__Cyanobacteria;c__Synechococcophycideae;o__Synechococcales;f__Acaryochloridaceae;g__Acaryochloris | 0.38 |
| 47 | p__Firmicutes;c__Bacilli;o__Bacillales;f__Alicyclobacillaceae;g__Alicyclobacillus | 0.02 |
| 48 | p__Firmicutes;c__Bacilli;o__Bacillales;f__Bacillaceae;g__Bacillus | 0.41 |
| 49 | p__Firmicutes;c__Bacilli;o__Bacillales;f__Paenibacillaceae;g__Brevibacillus | 0.04 |
| 50 | p__Firmicutes;c__Bacilli;o__Bacillales;f__Paenibacillaceae;g__Paenibacillus | 0.05 |
| 51 | p__Firmicutes;c__Bacilli;o__Bacillales;f__Planococcaceae;g__Sporosarcina | 0.03 |
| 52 | p__Firmicutes;c__Bacilli;o__Bacillales;f__Staphylococcaceae;g__Staphylococcus | 0.44 |
| 53 | p__Firmicutes;c__Clostridia;o__Clostridiales;f__[Tissierellaceae];g__Anaerococcus | 0.12 |
| 54 | p__Firmicutes;c__Clostridia;o__Clostridiales;f__[Tissierellaceae];g__Peptoniphilus | 0.11 |
| 55 | p__Planctomycetes;c__Planctomycetia;o__Gemmatales;f__Gemmataceae;g__Gemmata | 0.05 |
| 56 | p__Planctomycetes;c__Planctomycetia;o__Planctomycetales;f__Planctomycetaceae;g__Planctomyces | 0.05 |
| 57 | p__Proteobacteria;c__Alphaproteobacteria;o__Caulobacterales;f__Caulobacteraceae;g__Phenylobacterium | 0.08 |
| 58 | p__Proteobacteria;c__Alphaproteobacteria;o__Rhizobiales;f__Bradyrhizobiaceae;g__Balneimonas | 0.20 |
| 59 | p__Proteobacteria;c__Alphaproteobacteria;o__Rhizobiales;f__Hyphomicrobiaceae;g__Devosia | 0.27 |
| 60 | p__Proteobacteria;c__Alphaproteobacteria;o__Rhizobiales;f__Hyphomicrobiaceae;g__Hyphomicrobium | 0.10 |
| 61 | p__Proteobacteria;c__Alphaproteobacteria;o__Rhizobiales;f__Hyphomicrobiaceae;g__Pedomicrobium | 0.13 |
| 62 | p__Proteobacteria;c__Alphaproteobacteria;o__Rhizobiales;f__Hyphomicrobiaceae;g__Rhodoplanes | 0.60 |
| 63 | p__Proteobacteria;c__Alphaproteobacteria;o__Rhizobiales;f__Methylobacteriaceae;g__Methylobacterium | 0.60 |
| 64 | p__Proteobacteria;c__Alphaproteobacteria;o__Rhizobiales;f__Phyllobacteriaceae;g__Mesorhizobium | 0.09 |
| 65 | p__Proteobacteria;c__Alphaproteobacteria;o__Rhizobiales;f__Rhizobiaceae;g__Agrobacterium | 0.62 |
| 66 | p__Proteobacteria;c__Alphaproteobacteria;o__Rhizobiales;f__Rhodobiaceae;g__Afifella | 0.03 |
| 67 | p__Proteobacteria;c__Alphaproteobacteria;o__Rhodobacterales;f__Rhodobacteraceae;g__Paracoccus | 0.27 |
| 68 | p__Proteobacteria;c__Alphaproteobacteria;o__Rhodobacterales;f__Rhodobacteraceae;g__Rubellimicrobium | 0.20 |
| 69 | p__Proteobacteria;c__Alphaproteobacteria;o__Rhodospirillales;f__Acetobacteraceae;g__Roseococcus | 0.01 |
| 70 | p__Proteobacteria;c__Alphaproteobacteria;o__Rhodospirillales;f__Rhodospirillaceae;g__Azospirillum | 0.03 |
| 71 | p__Proteobacteria;c__Alphaproteobacteria;o__Rhodospirillales;f__Rhodospirillaceae;g__Skermanella | 0.12 |
| 72 | p__Proteobacteria;c__Alphaproteobacteria;o__Rickettsiales;f__Rickettsiaceae;g__Rickettsia | 23.54 |
| 73 | p__Proteobacteria;c__Alphaproteobacteria;o__Rickettsiales;f__Rickettsiaceae;g__Wolbachia | 0.90 |
| 74 | p__Proteobacteria;c__Alphaproteobacteria;o__Sphingomonadales;f__Sphingomonadaceae;g__Kaistobacter | 1.62 |
| 75 | p__Proteobacteria;c__Alphaproteobacteria;o__Sphingomonadales;f__Sphingomonadaceae;g__Novosphingobium | 0.08 |
| 76 | p__Proteobacteria;c__Betaproteobacteria;o__Burkholderiales;f__Comamonadaceae;g__Delftia | 0.47 |
| 77 | p__Proteobacteria;c__Betaproteobacteria;o__Tremblayales;f__Tremblayaceae;g__Candidatus Tremblaya | 0.48 |
| 78 | p__Proteobacteria;c__Deltaproteobacteria;o__Bdellovibrionales;f__Bdellovibrionaceae;g__Bdellovibrio | 0.04 |
| 79 | p__Proteobacteria;c__Gammaproteobacteria;o__Alteromonadales;f__[Chromatiaceae];g__Rheinheimera | 0.04 |
| 80 | p__Proteobacteria;c__Gammaproteobacteria;o__Enterobacteriales;f__Enterobacteriaceae;g__Candidatus Hamiltonella | 7.02 |
| 81 | p__Proteobacteria;c__Gammaproteobacteria;o__Oceanospirillales;f__Halomonadaceae;g__Candidatus Portiera | 52.37 |
| 82 | p__Proteobacteria;c__Gammaproteobacteria;o__Pseudomonadales;f__Moraxellaceae;g__Acinetobacter | 0.38 |
| 83 | p__Proteobacteria;c__Gammaproteobacteria;o__Pseudomonadales;f__Pseudomonadaceae;g__Pseudomonas | 0.20 |
| 84 | p__Proteobacteria;c__Gammaproteobacteria;o__Xanthomonadales;f__Sinobacteraceae;g__Steroidobacter | 0.05 |
| 85 | p__Proteobacteria;c__Gammaproteobacteria;o__Xanthomonadales;f__Xanthomonadaceae;g__Dokdonella | 0.03 |
| 86 | p__Proteobacteria;c__Gammaproteobacteria;o__Xanthomonadales;f__Xanthomonadaceae;g__Lysobacter | 0.09 |
| 87 | p__Proteobacteria;c__Gammaproteobacteria;o__Xanthomonadales;f__Xanthomonadaceae;g__Stenotrophomonas | 0.20 |
| 88 | p__Verrucomicrobia;c__Opitutae;o__Opitutales;f__Opitutaceae;g__Opitutus | 0.03 |
| 89 | p__Verrucomicrobia;c__[Spartobacteria];o__[Chthoniobacterales];f__[Chthoniobacteraceae];g__DA101 | 0.03 |
| 90 | p__[Thermi];c__Deinococci;o__Deinococcales;f__Deinococcaceae;g__Deinococcus | 0.22 |
| 91 | p__[Thermi];c__Deinococci;o__Deinococcales;f__Trueperaceae;g__Truepera | 0.15 |
| **CATONGO** | | |
| 1 | p__Acidobacteria;c__Solibacteres;o__Solibacterales;f__Solibacteraceae;g__Candidatus Solibacter | 0.05 |
| 2 | p__Actinobacteria;c__Actinobacteria;o__Actinomycetales;f__Actinomycetaceae;g__Actinobaculum | 0.02 |
| 3 | p__Actinobacteria;c__Actinobacteria;o__Actinomycetales;f__Brevibacteriaceae;g__Brevibacterium | 0.02 |
| 4 | p__Actinobacteria;c__Actinobacteria;o__Actinomycetales;f__Cellulomonadaceae;g__Cellulomonas | 0.01 |
| 5 | p__Actinobacteria;c__Actinobacteria;o__Actinomycetales;f__Corynebacteriaceae;g__Corynebacterium | 0.05 |
| 6 | p__Actinobacteria;c__Actinobacteria;o__Actinomycetales;f__Dermabacteraceae;g__Brachybacterium | 0.22 |
| 7 | p__Actinobacteria;c__Actinobacteria;o__Actinomycetales;f__Dermacoccaceae;g__Dermacoccus | 0.01 |
| 8 | p__Actinobacteria;c__Actinobacteria;o__Actinomycetales;f__Microbacteriaceae;g__Curtobacterium | 0.04 |
| 9 | p__Actinobacteria;c__Actinobacteria;o__Actinomycetales;f__Microbacteriaceae;g__Leucobacter | 0.02 |
| 10 | p__Actinobacteria;c__Actinobacteria;o__Actinomycetales;f__Microbacteriaceae;g__Microbacterium | 0.02 |
| 11 | p__Actinobacteria;c__Actinobacteria;o__Actinomycetales;f__Micrococcaceae;g__Kocuria | 0.06 |
| 12 | p__Actinobacteria;c__Actinobacteria;o__Actinomycetales;f__Micrococcaceae;g__Nesterenkonia | 0.24 |
| 13 | p__Actinobacteria;c__Actinobacteria;o__Actinomycetales;f__Micromonosporaceae;g__Actinoplanes | 0.02 |
| 14 | p__Actinobacteria;c__Actinobacteria;o__Actinomycetales;f__Mycobacteriaceae;g__Mycobacterium | 0.05 |
| 15 | p__Actinobacteria;c__Actinobacteria;o__Actinomycetales;f__Nocardioidaceae;g__Nocardioides | 0.03 |
| 16 | p__Actinobacteria;c__Actinobacteria;o__Actinomycetales;f__Propionibacteriaceae;g__Propionibacterium | 0.33 |
| 17 | p__Actinobacteria;c__Actinobacteria;o__Actinomycetales;f__Pseudonocardiaceae;g__Actinomycetospora | 0.30 |
| 18 | p__Actinobacteria;c__Actinobacteria;o__Actinomycetales;f__Pseudonocardiaceae;g__Pseudonocardia | 0.42 |
| 19 | p__Actinobacteria;c__Actinobacteria;o__Actinomycetales;f__Streptomycetaceae;g__Streptomyces | 0.04 |
| 20 | p__Actinobacteria;c__Rubrobacteria;o__Rubrobacterales;f__Rubrobacteraceae;g__Rubrobacter | 0.33 |
| 21 | p__Bacteroidetes;c__Bacteroidia;o__Bacteroidales;f__Porphyromonadaceae;g__Dysgonomonas | 1.50 |
| 22 | p__Bacteroidetes;c__Cytophagia;o__Cytophagales;f__Cytophagaceae;g__Adhaeribacter | 0.05 |
| 23 | p__Bacteroidetes;c__Cytophagia;o__Cytophagales;f__Cytophagaceae;g__Hymenobacter | 0.22 |
| 24 | p__Bacteroidetes;c__Cytophagia;o__Cytophagales;f__Cytophagaceae;g__Pontibacter | 0.04 |
| 25 | p__Bacteroidetes;c__Cytophagia;o__Cytophagales;f__Cytophagaceae;g__Rhodocytophaga | 0.05 |
| 26 | p__Bacteroidetes;c__Cytophagia;o__Cytophagales;f__Cytophagaceae;g__Spirosoma | 0.09 |
| 27 | p__Bacteroidetes;c__Flavobacteriia;o__Flavobacteriales;f__[Weeksellaceae];g__Chryseobacterium | 0.03 |
| 28 | p__Bacteroidetes;c__Sphingobacteriia;o__Sphingobacteriales;f__Sphingobacteriaceae;g__Sphingobacterium | 0.04 |
| 29 | p__Bacteroidetes;c__[Saprospirae];o__[Saprospirales];f__Chitinophagaceae;g__Flavisolibacter | 0.14 |
| 30 | p__Chloroflexi;c__Anaerolineae;o__Ardenscatenales;f__Ardenscatenaceae;g__Ardenscatena | 0.04 |
| 31 | p__Cyanobacteria;c__Nostocophycideae;o__Nostocales;f__Scytonemataceae;g__Brasilonema | 0.07 |
| 32 | p__Cyanobacteria;c__Nostocophycideae;o__Nostocales;f__Scytonemataceae;g__Scytonema | 0.12 |
| 33 | p__Cyanobacteria;c__Nostocophycideae;o__Stigonematales;f__Rivulariaceae;g__Calothrix | 0.60 |
| 34 | p__Cyanobacteria;c__Oscillatoriophycideae;o__Chroococcales;f__Xenococcaceae;g__Chroococcidiopsis | 0.09 |
| 35 | p__Cyanobacteria;c__Oscillatoriophycideae;o__Oscillatoriales;f__Phormidiaceae;g__Microcoleus | 0.03 |
| 36 | p__Cyanobacteria;c__Oscillatoriophycideae;o__Oscillatoriales;f__Phormidiaceae;g__Phormidium | 0.06 |
| 37 | p__Cyanobacteria;c__Synechococcophycideae;o__Pseudanabaenales;f__Pseudanabaenaceae;g__Leptolyngbya | 0.36 |
| 38 | p__Cyanobacteria;c__Synechococcophycideae;o__Synechococcales;f__Acaryochloridaceae;g__Acaryochloris | 0.13 |
| 39 | p__Firmicutes;c__Bacilli;o__Bacillales;f__Bacillaceae;g__Bacillus | 0.34 |
| 40 | p__Firmicutes;c__Bacilli;o__Bacillales;f__Planococcaceae;g__Sporosarcina | 0.02 |
| 41 | p__Firmicutes;c__Bacilli;o__Bacillales;f__Staphylococcaceae;g__Staphylococcus | 0.22 |
| 42 | p__Firmicutes;c__Bacilli;o__Lactobacillales;f__Enterococcaceae;g__Enterococcus | 0.02 |
| 43 | p__Firmicutes;c__Bacilli;o__Lactobacillales;f__Streptococcaceae;g__Lactococcus | 0.08 |
| 44 | p__Planctomycetes;c__Planctomycetia;o__Gemmatales;f__Gemmataceae;g__Gemmata | 0.05 |
| 45 | p__Planctomycetes;c__Planctomycetia;o__Planctomycetales;f__Planctomycetaceae;g__Planctomyces | 0.05 |
| 46 | p__Proteobacteria;c__Alphaproteobacteria;o__Rhizobiales;f__Bradyrhizobiaceae;g__Balneimonas | 0.10 |
| 47 | p__Proteobacteria;c__Alphaproteobacteria;o__Rhizobiales;f__Hyphomicrobiaceae;g__Devosia | 0.20 |
| 48 | p__Proteobacteria;c__Alphaproteobacteria;o__Rhizobiales;f__Hyphomicrobiaceae;g__Pedomicrobium | 0.05 |
| 49 | p__Proteobacteria;c__Alphaproteobacteria;o__Rhizobiales;f__Hyphomicrobiaceae;g__Rhodoplanes | 0.33 |
| 50 | p__Proteobacteria;c__Alphaproteobacteria;o__Rhizobiales;f__Methylobacteriaceae;g__Methylobacterium | 0.49 |
| 51 | p__Proteobacteria;c__Alphaproteobacteria;o__Rhizobiales;f__Rhizobiaceae;g__Agrobacterium | 0.61 |
| 52 | p__Proteobacteria;c__Alphaproteobacteria;o__Rhodobacterales;f__Rhodobacteraceae;g__Paracoccus | 0.10 |
| 53 | p__Proteobacteria;c__Alphaproteobacteria;o__Rhodobacterales;f__Rhodobacteraceae;g__Rubellimicrobium | 0.23 |
| 54 | p__Proteobacteria;c__Alphaproteobacteria;o__Rhodospirillales;f__Rhodospirillaceae;g__Skermanella | 0.04 |
| 55 | p__Proteobacteria;c__Alphaproteobacteria;o__Rickettsiales;f__Rickettsiaceae;g__Rickettsia | 23.70 |
| 56 | p__Proteobacteria;c__Alphaproteobacteria;o__Rickettsiales;f__Rickettsiaceae;g__Wolbachia | 1.13 |
| 57 | p__Proteobacteria;c__Alphaproteobacteria;o__Sphingomonadales;f__Sphingomonadaceae;g__Kaistobacter | 0.91 |
| 58 | p__Proteobacteria;c__Alphaproteobacteria;o__Sphingomonadales;f__Sphingomonadaceae;g__Novosphingobium | 0.12 |
| 59 | p__Proteobacteria;c__Alphaproteobacteria;o__Sphingomonadales;f__Sphingomonadaceae;g__Sphingobium | 0.03 |
| 60 | p__Proteobacteria;c__Alphaproteobacteria;o__Sphingomonadales;f__Sphingomonadaceae;g__Sphingomonas | 1.33 |
| 61 | p__Proteobacteria;c__Betaproteobacteria;o__Burkholderiales;f__Burkholderiaceae;g__Burkholderia | 0.03 |
| 62 | p__Proteobacteria;c__Betaproteobacteria;o__Burkholderiales;f__Comamonadaceae;g__Delftia | 0.44 |
| 63 | p__Proteobacteria;c__Betaproteobacteria;o__Tremblayales;f__Tremblayaceae;g__Candidatus Tremblaya | 0.35 |
| 64 | p__Proteobacteria;c__Gammaproteobacteria;o__Alteromonadales;f__[Chromatiaceae];g__Rheinheimera | 0.04 |
| 65 | p__Proteobacteria;c__Gammaproteobacteria;o__Enterobacteriales;f__Enterobacteriaceae;g__Candidatus Hamiltonella | 9.73 |
| 66 | p__Proteobacteria;c__Gammaproteobacteria;o__Oceanospirillales;f__Halomonadaceae;g__Candidatus Portiera | 52.50 |
| 67 | p__Proteobacteria;c__Gammaproteobacteria;o__Pseudomonadales;f__Moraxellaceae;g__Acinetobacter | 0.26 |
| 68 | p__Proteobacteria;c__Gammaproteobacteria;o__Pseudomonadales;f__Moraxellaceae;g__Perlucidibaca | 0.02 |
| 69 | p__Proteobacteria;c__Gammaproteobacteria;o__Pseudomonadales;f__Pseudomonadaceae;g__Pseudomonas | 0.09 |
| 70 | p__Proteobacteria;c__Gammaproteobacteria;o__Xanthomonadales;f__Xanthomonadaceae;g__Dokdonella | 0.02 |
| 71 | p__Verrucomicrobia;c__[Spartobacteria];o__[Chthoniobacterales];f__[Chthoniobacteraceae];g__Ellin506 | 0.01 |
| 72 | p__[Thermi];c__Deinococci;o__Deinococcales;f__Deinococcaceae;g__Deinococcus | 0.33 |
| 73 | p__[Thermi];c__Deinococci;o__Deinococcales;f__Trueperaceae;g__Truepera | 0.11 |

Classification was determined for the two biological samples and six technical replicates of each genotype (CCN51 and Catongo).
