# Supplementary material for: Witches’ broom resistant genotype CCN51 shows greater diversity of symbiont bacteria in its phylloplane than susceptible genotype catongo
Source: BMC Microbiol. 2018 Nov 23;18:194. doi: 10.1186/s12866-018-1339-9 (PMC6251189; doi:10.1186/s12866-018-1339-9)
Supplement: Supplementary file 5 — Table S2. Bacterial genera exclusive to the phylloplane of the genotypes CCN51 and Catongo. (DOCX 17 kb) [file 12866_2018_1339_MOESM5_ESM.docx]

**Table S2.** Bacterial genera exclusive to the filoplano of the genotypes CCN51 and Catongo, with a threshold of 99% identity against the Greengen database version 13.8 16S rRNA.

| **CCN51** | | |
| --- | --- | --- |
| **Nº** | **Genus** | **Percentage** |
| 1 | *Actinomadura* | 1.13 |
| 2 | *Afifella* | 2.25 |
| 3 | *Alicyclobacillus* | 1.50 |
| 4 | *Anaerococcus* | 7.79 |
| 5 | *Arthrobacter* | 1.70 |
| 6 | *Azospirillum* | 1.86 |
| 7 | *Bdellovibrio* | 2.36 |
| 8 | *Brevibacillus* | 2.59 |
| 9 | *Chloronema* | 0.85 |
| 10 | *DA101* | 2.04 |
| 11 | *Fimbriimonas* | 2.62 |
| 12 | *Flavobacterium* | 2.13 |
| 13 | *Hyphomicrobium* | 6.55 |
| 14 | *Kineococcus* | 0.92 |
| 15 | *Kribbella* | 1.36 |
| 16 | *Lysobacter* | 5.89 |
| 17 | *Mesorhizobium* | 5.65 |
| 18 | *Micrococcus* | 2.32 |
| 19 | *Microlunatus* | 2.02 |
| 20 | *Nocardia* | 2.14 |
| 21 | *Opitutus* | 2.11 |
| 22 | *Oscillochloris* | 1.06 |
| 23 | *Paenibacillus* | 3.13 |
| 24 | *Peptoniphilus* | 7.04 |
| 25 | *Phenylobacterium* | 5.01 |
| 26 | *Pilimelia* | 1.67 |
| 27 | *Rhodococcus* | 1.68 |
| 28 | *Roseococcus* | 0.97 |
| 29 | *Rubricoccus* | 2.22 |
| 30 | *Sporocytophaga* | 1.73 |
| 31 | *Stenotrophomonas* | 13.55 |
| 32 | *Steroidobacter* | 3.05 |
| 33 | *Xylanimicrobium* | 1.11 |
| **CATONGO** | | |
| 1 | *Ardenscatena* | 1.90 |
| 2 | *Burkholderia* | 1.67 |
| 3 | *Cellulomonas* | 0.79 |
| 4 | *Chryseobacterium* | 1.54 |
| 5 | *Dermacoccus* | 1.00 |
| 6 | *Ellin506* | 1.00 |
| 7 | *Enterococcus* | 1.37 |
| 8 | *Lactococcus* | 3.88 |
| 9 | *Microcoleus* | 2.03 |
| 10 | *Perlucidibaca* | 1.50 |
| 11 | *Phormidium* | 3.87 |
| 12 | *Pontibacter* | 2.34 |
| 13 | *Sphingobacterium* | 1.81 |
| 14 | *Sphingomonas* | 75.30 |

Classification was determined for the two biological samples and six technical replicates of each genotype (CCN51 and Catongo).
